# Response to Letter—Qualitative Exploration of the Barriers and Facilitators to Early Lower Limb Assessment and Onward Referral for Specialist Treatment for Patients With Venous Ulceration

**DOI:** 10.1111/iwj.70493

**Published:** 2025-04-06

**Authors:** Layla Bolton Saghdaoui, Smaragda Lampridou, Alun Huw Davies, Sarah Onida, Mary Wells

**Affiliations:** ^1^ Section of Vascular Surgery – Department of Surgery and Cancer Imperial College London/Imperial College Healthcare NHS Trust, Charing Cross Hospital London UK; ^2^ Imperial College Healthcare NHS Trust/Imperial College London, Education Centre, Charing Cross Hospital London UK

Dear Authors,

We would like to thank you for taking the time to read and provide insightful comments on our paper. This qualitative study served as preliminary work conducted as part of a pre‐doctoral project. As noted in our study limitations, we very much agree with the need to integrate behavioural theory and provide policy recommendations, however further work is needed to do this comprehensively. This work is currently underway in the form of a mixed methods PhD project funded by the National Institute of Health and Care Research (NIHR) [1]. Building on the findings of this study and the qualitative evaluation of the implementation of the National Wound Care strategy [2], this project follows the Person‐Based Approach [3] and encompasses the following:

1. A national survey of healthcare professionals across the UK evaluating current referral practices and services available to treat patients with venous ulceration. This was supported by a simplified version of the survey sent to every Trust in the country as a freedom of information request (Manuscript under review—27/01/2025).
2. A systematic review of interventions to improve referral for chronic health conditions (Accepted for publication in BMC Systematic Reviews—07/Feb/2025).
3. A larger UK qualitative study informed by the Theoretical Domains Framework [4] and including a broader range of healthcare professionals, patients and their caregivers/significant others.
4. A planned Delphi study to refine data gathered in previous work packages and provide robust policy and practice recommendations.

Findings from the national survey and systematic review have been presented at recent national and international conferences. We look forward to disseminating further work over the coming months.

We would be happy to explore the possibility of potential collaboration should the authors be interested.
